# Effects of Dietary Supplementation in Patients with Restless Legs Syndrome: A Systematic Review

**DOI:** 10.3390/nu16142315

**Published:** 2024-07-18

**Authors:** Pedro González-Parejo, Javier Martín-Núñez, Irene Cabrera-Martos, Marie Carmen Valenza

**Affiliations:** Department of Physical Therapy, Faculty of Health Sciences, University of Granada, 18016 Granada, Spain; gppedro@correo.ugr.es (P.G.-P.); javimn@ugr.es (J.M.-N.); cvalenza@ugr.es (M.C.V.)

**Keywords:** restless legs syndrome, dietary supplements, nutrition therapy

## Abstract

Restless legs syndrome (RLS) is a common neurological disorder. It disrupts sleep and well-being and is often associated with other conditions. This review examines the potential of dietary supplements to manage RLS symptoms and reduce reliance on medications. A total of 10 randomized clinical trials involving 482 participants were analyzed, focusing on the impact of various supplements on symptom severity, sleep quality, and daytime sleepiness. Findings suggest some supplements may have positive results. Magnesium oxide and vitamin B6 significantly improved sleep quality and RLS symptoms, with magnesium showing greater effectiveness. Vitamin D supplementation did not show significant benefits. Oral iron has promising results, indicating potential efficacy but issues related to compliance and absorption. Both vitamins C and E positively affect RLS symptoms, likely due to their antioxidant properties. Valerian improved RLS and sleep but did not show a statistically significant improvement. Despite these encouraging results, a high risk of bias was noted in half of the studies, emphasizing the need for more rigorous research. Overall, this review suggests that dietary supplements may be a promising approach to managing RLS. However, further investigation is required to confirm the efficacy and safety.

## 1. Introduction

Restless legs syndrome (RLS) or Willis–Ekbom disease (WED), is a movement disorder that frequently exerts a significant influence on the quality of sleep. The intensity of the symptoms exhibits considerable variation, spanning from occasional manifestation in stressful circumstances to nightly and severe occurrences, causing nearly complete disruption of sleep [1]. It received its name because of Sir Thomas Willis (the first one that considered this syndrome) and Karl Ekbom several centuries after, who were the ones who first described this condition [2,3].

The most widely used diagnostic criteria are those proposed by the International Restless Legs Syndrome Study Group (IRLSSG) in its 2003 version [4]. The most current version is from 2014, where RLS is defined as a neurological sensorimotor disorder that frequently disturbs sleep and quality of life. This syndrome is diagnosed by identifying symptom patterns that meet five essential criteria and using clinical specifiers afterward to differentiate between a chronic persistent and intermittent pattern [5]. These essential diagnostic criteria include the following: (1) an urge to move the legs usually but not always accompanied by or felt to be caused by uncomfortable and unpleasant sensations in the legs, (2) the urge to move the legs and any accompanying unpleasant sensations begin or worsen during periods of rest or inactivity such as lying down or sitting, (3) the urge to move the legs and any accompanying unpleasant sensations are partially or relieved by movement, such as walking or stretching, at least as long as the activity continues, (4) the urge to move the legs and any accompanying unpleasant sensations during rest or inactivity only occur or are worse in the evening or night than during the day, (5) the occurrence of the above features is not solely accounted for as symptoms primary to another medical or a behavioral condition.

The estimated prevalence of RLS in the general population, according to various epidemiological studies, ranges from 1 to 16%, being more prevalent in American and European Caucasians rather than Asians and other non-Caucasian populations [4,6,7]. Furthermore, the prevalence increases in the presence of other pathological conditions such as kidney diseases, iron deficiency, cardiovascular diseases, diabetes, neuropathies, migraines, pregnant women, and patients with Parkinson’s disease undergoing dopaminergic treatment [8,9]. Although the pathophysiology of RLS is not fully understood, the most accepted pathways include genetic variants, abnormal iron metabolisms, dopaminergic dysfunction, and central opiate system, which could influence its clinical development [10,11]. The initial and foremost factors to consider in addressing RLS encompass the assessment of iron levels, the clinical significance of symptoms, coexisting sleep disorders, and the role of medications exacerbating its symptoms [12,13]. The therapy usually starts when symptoms influence the patient’s quality of life [14], and according to the clinical severity of each case, the selection of a specific therapy will be determined.

When dealing with moderate to severe, chronic, or persistent RLS, it usually requires a pharmacological intervention [12] focused on symptomatology relief [15]. In addition, patients with mild intermittent symptoms can often be managed by non-pharmacological approaches and lifestyle modifications. It is also worth noticing that the interventions should also be considered complementary therapy when using other pharmacological treatments [12,16].

Although pharmacological therapy has been studied more thoroughly than non-pharmacological [17], it is only needed when symptoms exhibit clinical significance with quality-of-life impairment, which happens in about 10 to 15% of RLS patients [18,19]. In this context, non-pharmacological therapies would facilitate a drug utilization decrease and the occurrence of adverse effects such as augmentation, defined as a symptom worsening attributable to the long-term use of dopaminergic medication [20].

Among the various non-pharmacological therapies, dietary supplementation emerges as a notable option. According to the Official Journal of European Communities, dietary supplements are concentrated sources of nutrients or other substances with a nutritional or physiological effect, alone or in combination, marketed in dose form, namely forms such as capsules, pastilles, tablets, pills, and other similar forms, sachets of powder, ampoules of liquids, drop-dispensing bottles, and other similar forms of liquids and powders designed to be taken in measured small unit quantities [21]. Dietary supplements include vitamins, minerals, herbs or other botanicals and amino acids [22]. The physiological rationale for utilizing dietary supplements for RLS treatment stems from the theory that oxidative stress and the consequent damage from free radicals in the central nervous system affect the balance of iron in the brain, thereby affecting dopamine synthesis, since iron is essential for tyrosine hydroxylase, a dopamine precursor. This theory finds support in the observed link between iron deficiency and secondary RLS, as well as the effectiveness of dopamine agonist therapy as a primary pharmacological intervention for managing RLS symptoms in a significant portion of patients [23]. Magnesium supplementation is widely used for nocturnal leg cramps, which has been proposed to be associated with magnesium deficiency, and it could be effective in alleviating RLS symptoms as it is found to be biologically plausible [24]. Several observational studies have also reported an association between lower magnesium levels and the severity of RLS [25,26]. There is also a high prevalence of vitamin D deficiency in people with RLS, where vitamin D supplementation seems to be responsive [27]. Low levels of vitamin C and vitamin E are among the various factors contributing to high levels of oxidative stress among hemodialysis patients [28]. Vitamin C has been demonstrated to have therapeutic effects on sleep disturbances and sensorimotor abnormalities such as leg cramps [29]. Furthermore, there are reports on the efficacy of vitamin E in idiopathic RLS [30,31]. Lastly, valerian has been indicated for the treatment of sleep problems related to anxiety or lack of rest, which could be an alternative option to pharmaceutical drug treatment in RLS patients [32].

Currently, there is limited evidence assessing the impact of oral supplementation in patients with RLS [23,25,33], and does not consider all the supplements available. Therefore, the objective of this systematic review was to evaluate the effect of dietary supplementation on the clinical severity of patients with RLS.

## 2. Materials and Methods

### 2.1. Eligibility Criteria

The review included only randomized clinical trials that evaluated the impact of dietary supplementation on the clinical severity of people diagnosed with RLS. There were no limitations in terms of publication date, and articles written in English, Spanish, French, Italian, Brazilian, and Portuguese were considered eligible for selection. Studies conducted in animals or pediatric patients were excluded, and regarding the patient’s profile, no other restrictions were defined in terms of sex, diagnostic criteria, other concomitant pathologies, or among different variants of RLS.

### 2.2. Search Strategy

We have conducted a systematic review according to the guidelines outlined in the PRISMA statement [34] and registered at PROSPERO (ID: CRD42024506679). As a guide for the study of the systematic review, the following PICOS question is posed (P: Adults diagnosed with RLS, I: Dietary supplements intake, C: Patients not taking dietary supplements or taking placebo, O: Clinical severity, S: Randomized clinical trials): “What effect could the use of dietary supplementation have on the prevalence and clinical severity of restless legs syndrome?”.

The search was performed in February 2024 across PubMed, Scopus, Web of Science, CINAHL, and Science Direct databases. Title and abstract screening were initially conducted to identify relevant articles. Subsequently, the remaining articles were full-text reviewed for a deeper analysis. Two authors independently reviewed the articles. Unreleased materials or abstracts were not considered in this systematic review.

### 2.3. Data Extraction

Information was methodically gathered to fulfill the objectives of the review. The following data were extracted: author(s) and year of publication, sample size, groups compared, inclusion and exclusion criteria, participant’s age, female presence, duration of the trial and number of measurements, outcome measurements, and patients lost in follow-up.

The main variable of this systematic review was the improvement in symptom severity, and secondary variables related to sleep quality and daytime sleepiness were also collected. All information regarding the data extracted from the articles included in this systematic review is detailed in Table 1 and Table 2.

### 2.4. Quality Assessment

To assess the risk of bias, we adhered to the “Revised Cochrane risk-of-bias tool for randomized trials” (RoB2) for randomized studies, as it is endorsed by the Cochrane Handbook for Systematic Reviews of Interventions and represents the latest guideline for assessing bias risk in randomized clinical trials [35]. RoB2 is the recommended tool for assessing this risk of bias and is organized into specific bias domains, which examine various aspects of trial planning, conduct, and reporting. These domains include the randomization process, deviations from intended interventions, missing outcome data, outcome measurement, and selection of the reported result. Each domain contains a set of questions designed to collect details about trial characteristics that affect the risk of bias. An algorithm processes the responses to these questions to evaluate the risk of bias in each domain. The assessment can result in categorizations such as “Low”, “Some concerns”, or “High” risk of bias [35].

**Table 1:** Characteristics of selected studies.

Studies	Sample (n)	Sample Size; Age (years ± SD); and % Women				Loss to Follow-Up	Inclusion and Exclusion Criteria
Haiizadeh et al. (2023) [36]	40	Valerian (20) 59 ± 15 56%	Gabapentin (20) 60 ± 15 53%			9 (23%)	IC: Score > 10 on the RLS questionnaire or >5 on the PSQI; >14 years, BMI < 30 and blood pressure > 110/70. EC: Allergy to valerian, having a physical disability, mental disorders, treatment with psychiatric medications, deafness or blindness, pregnancy, and having taken Gabapentin routinely.
Jadidi et al. (2022) [37]	75	Vit B6 (25) 38 ± 8 72%	Mg (25) 41 ± 7 80%	* Placebo (25) 40 ± 10 76%		0	IC: 3-month history of RLS, 15–50 years and no risk factors. EC: Reluctance to continue the trial, pregnancy during the study and adverse medication reactions.
Wali et al. (2019) [38]	35	Vit D (17) 43 ± 5 35%	** Placebo (18) 42 ± 5 28%			13 (37%)	IC: Adults with primary RLS who were not being treated for RLS or receiving vitamin D EC: Disorders that mimic RLS, medications that could trigger RLS or interfere with vitamin D absorption, contraindications to vitamin D, use of supplements containing vitamin D or calcium, history of vitamin D intolerance, pregnancy, lactation, or oral contraceptives.
Vishwalarma et al. (2016) [39]	90	Bupropion (30) 39 ± 12 67%	Ropinirol (30) 44 ± 14 67%	Fe + Vit B9 (30) 38 ± 12 77%		13 (13%)	IC: Adults who attended the sleep clinic and were diagnosed with RLS according to the IRLS. EC: Comorbidities, opioid/opioid use or experiencing opioid withdrawal, alcohol dependence, use of neuroleptics, antidepressants, antiparkinsonian medications, uremia or end-stage renal disease, obstructive sleep apnea, parasomnia, hypersomnia and neurocognitive disorders, pulmonary disease, or pregnant women.
Rafie et al. (2016) [40]	44	Vit C (15) 53 ± 10 67%	Pramipexole (14) 57 ± 13 64%	* Placebo (15) 58 ± 8 33%		1 (2%)	IC: 18–80 years old regularly undergoing hemodialysis and diagnosed according to IRLS. EC: Unstable vital signs, affected by acute illness; tricyclic antidepressants, selective serotonin reuptake inhibitors, dopamine antagonists, dopamine blocking antiemetics, lithium and hypnotic antihistamines, history of renal stones.
Lee et al. (2014) [41]	30	Fe (15) 53 ± 13 93%	Pramipexole (15) 59 ± 11 100%			7 (23%)	IC: 20–80 years old, diagnosis of RLS and a serum ferritin concentration between 15 and 50 ng/mL. EC: Pregnant women, history of hemochromatosis, severe liver disease, end-stage renal disease or malignancy, iron allergy and intake of iron supplements or medications that would affect RLS symptoms.
Sagheb et al. (2012) [29]	60	Vit C + Vit E (15) 47 ± 14 60%	Vit C + Placebo (15) 55 ± 15 60%	Vit E + Placebo (15) 49 ± 12 73%	** Placebo (15) 59 ± 18 40%	0	IC: 18–80 years, stable patients on regular hemodialysis without acute illness or hospitalization, with all IRLS diagnostic criteria. EC: Medications with properties that aggravate RLS or history of kidney stones.
Cuellar et al. (2009) [32]	48	Valerian (24) 50 ± 13 71%	^a^ Placebo (24) 49 ± 13 79%			11 (23%)	IC: >21 years old, with RLS and akathisia, with symptoms 3 nights a week or more. EC: Positive toxicology report, abnormal liver function profile and 3 affirmative responses on the CAGE questionnaire. Participation in a clinical trial with an investigational drug in the last 3 months. Use of vitamins, minerals, herbal–natural products, benzodiazepines or barbiturates, valerian within the last 120 days, other sleep disorders. History of liver disease, including cirrhosis, alcoholism and hepatitis; pregnant, nursing or intending to become pregnant within the next 3 months.
Wang et al. (2009) [42]	18	Fe + Vit C (11) 60 (36–82) 54%	Vit C + ^c^ Placebo (7) 58 (33–72) 71%			0	IC: Diagnosis of RLS and ferritin levels of 15–75 ng/mL. EC: Pregnancy, hemochromatosis or other significant liver disease, end-stage renal disease, significant sleep disturbance, iron saturation less than 15%, hemoglobin levels < 11.1 g/dL for women and 14 g/dL for men, allergy to iron sulfate, current or recent treatment with iron sulfate.
Davis et al. (2000) [43]	28	Fe (14) 58.6 (33–80) 64%	^b^ Placebo (14) 60 (33–76) 71%			7 (25%)	IC: Symptomatic RLS under treatment EC: Iron sulfate allergy, anemia, pregnancy, hemochromatosis, peptic ulcer disease, history of gastrointestinal neoplasm within the last 2 years, active bacterial infection, or current treatment with medications known by patients to exacerbate their RLS.

Vit: Vitamin; Mg: Magnesium oxide; Fe: Oral iron; BMI: Body mass index; EC: Exclusion criteria; IC: Inclusion criteria; IRLS: International Restless Legs Syndrome Severity Scale; PSQI: Pittsburgh Sleep Quality Index; RLS: Restless Legs Syndrome; *: Not specified; **: Similar in size, color, weight and/or taste; ^a^: Lactose fillers; ^b^: H_2_O and 2% carboxymethylcellulose; ^c^: Lactose.

**Table 2:** Main findings of the included studies.

Studies	Supplementation, Dosage and Frequency			Comparison (Dose and Frequency)	Outcomes	Intervention Duration; Measuring Moments (Weeks)	Main Findings
Haiizadeh et al. (2023) [36]	Valerian 530 mg/day			Gabapentin 100 mg/day	IRLS PSQI	12; 0–4–8–12	Although both groups significantly reduced mean IRLS scores after the first phase, scores were lower in the gabapentin group. There was no statistically significant difference between the two groups in terms of sleep quality score. They concluded that gabapentin is more effective than valerian in improving IRLS, but both are equally effective in improving sleep quality.
Jadidi et al. (2022) [37]	Vit B6 40 mg/day	mg 200 mg/day		Placebo	IRLS PSQI	8; 0–4–8	Two months after the intervention, disease severity and sleep quality improved significantly in all three groups and were significantly different between groups (*p* < 0.05). Both intervention groups outperformed the control group and PSQI and IRLS scores were markedly lower in the magnesium oxide group.
Wali et al. (2019) [38]	Vit D 50,000 IU/week			Placebo	IRLS	12; 0–4–8–12	The vitamin D group did not show significant changes in IRLS scores from baseline to week 12 (*p* = 0.54), but they did in the placebo group (*p* = 0.04).
Vishwalarma et al. (2016) [39]	Ferrous sulfate 150 mg/day + Vit B9 500 µg/day			Propion (150 mg/day 5 days followed by 300 mg/day) or Ropinirole (0.25 mg/day 2 weeks, 0.5 mg/day thereafter)	IRLS RLS QoL	6; 0–2–4–6	IRLS scores differed significantly at all four measurement points from the initial to the last visit (*p* = 0.01). The interaction between time and treatment group was significant (*p* < 0.001), suggesting that all groups showed improvement with therapy. The three groups differed significantly from each other (*p* = 0.001). The RLS-related quality of life (RLS QoL) score at the baseline visit was significantly different from the other three visits (*p* < 0.001). Differences between groups were not significant (*p* = 0.28).
Rafie et al. (2016) [40]	Vit C 250 mg/day			Placebo o Pramipexole 0.18 mg/day	IRLS	8; 0–8	All three groups showed significant improvement. Both the vitamin C and pramipexole groups had significantly different scores compared to the placebo group; but their scores were not significantly different from each other (*p* = 0.77).
Lee et al. (2014) [41]	Ferrous sulfate 650 mg/day			Pramipexole 0.25 mg/day	IRLS PSQI ESS BDI	12; 0–2–4–8–12	Time was found to have a significant effect on IRLS score (*p* < 0.001), but no group effect (*p* = 0.99) or group-time interaction (*p* = 0.96) was observed. A significant reduction in PSQI score was observed in the pramipexole group, while no reduction was evident in the iron group. The ESS and BDI scales showed no significant changes.
Sagheb et al. (2012) [29]	Vit C 200 mg/day + Vit E 400 mg/day	Vit C 200 mg/day	Vitamin E 400 mg/day	Placebo	IRLS	8; 0–8	IRLS scores decreased in all groups at the end of treatment. Scores in each experimental group were significantly higher than in the placebo group (*p* < 0.001). However, there were no differences between treatment groups.
Cuellar et al. (2009) [32]	Valerian 800 mg/day			Placebo	IRLS PSQI ESS	8; 0–8	All patients experienced improvement in sleep quality and RLS severity. PSQI scores decreased in all components, as did total scores; these decreases were statistically significant (*p* < 0.05) for the first 5 components. The ESS also showed improvement in all subjects (*p* < 0.001). Changes in SPI scores between groups were not statistically different.
Wang et al. (2009) [42]	Ferrous sulfate 650 mg/day + Vit C 200 mg/day			Placebo + Vit C 200 mg/day	IRLS Quality of life	12; 0–6–12	The mean decrease in IRLS score after 12 weeks for the iron and placebo groups was 10 ± 7 and 1 ± 6, respectively, these differences being statistically significant (*p* = 0.01). When comparing dichotomized variables, a trend toward an improvement in quality of life was observed in both groups of (*p* = 0.07).
Davis et al. (2000) [43]	Ferrous sulfate 325 mg/day			Placebo	RLS Sleep RLS QoL	26; 0–2–4–14–26	No significant differences were observed between the iron and placebo groups for primary and secondary outcomes.

IRLS: International Restless Legs Syndrome Severity Scale; PSQI: Pittsburgh Sleep Quality Index; RLS QoL: Restless Legs Syndrome Related Quality of Life; ESS: Epworth Sleepiness Scale; BDI: Beck Depression Inventory.

The heterogeneity in the supplements used and in the inclusion and exclusion criteria, as well as a small number of records addressing each supplement specifically, did not allow a meta-analysis of the studied variables.

## 3. Results

### 3.1. Study Selection

**Figure 1:**
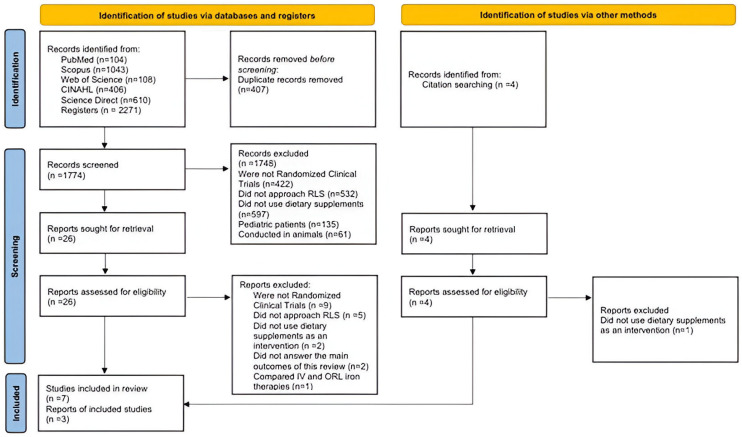
Preferred reporting items for systematic reviews and meta-analyses (PRISMA) flow chart for study inclusion.

The search retrieved a total of 2271 studies. After removing duplicates, 1774 titles and abstracts were screened. Studies were excluded if their methodology did not align with the research objective (e.g., not randomized clinical trials n = 422), study field (e.g., not addressing RLS n = 532), intervention (e.g., not using dietary supplements n = 597), or patient profile (e.g., pediatric patients n = 135; animal trials n = 61). Of the 26 remaining articles accessed for full-text reading, 19 were excluded: 9 for not being randomized clinical trials, 5 for not addressing RLS, 2 for not answering the review’s main outcome, 2 for not using supplements, and 1 for comparing intravenous (IV) with oral (ORL) iron. Finally, ten articles were included in this review; seven identified through the search strategy and screening [29,37,38,39,41,42,43], and three obtained from other sources [32,36,40]. The PRISMA flow chart is illustrated in Figure 1.

### 3.2. Study Characteristics

The characteristics of the selected studies are included in the Table 1. A total of 10 articles were included in this systematic review, with a total of 482 patients, out of which 233 took supplements as an intervention and 61 were lost during the follow-up. The sample size ranged across the trials between 18 [42] and 103 participants [39], and the median age ranged from 38 [39] to 60 years [36]. The duration of the follow-up in the included articles ranged from 6 [39] to 26 weeks [43], and the average percentage of female presence in the sample was approximately 63.65%. Among the various studies included in this review, a diverse range of dietary supplements was examined, such as vitamin B6 and magnesium oxide [37], vitamin D [38], vitamin C [29,40,42], vitamin E [29], oral iron [39,41,43], folic acid [39] and valerian [32,36].

The details regarding the supplementation dosage and frequency, outcomes and main findings are included in the Table 2.

### 3.3. Outcome Measures

The primary variable of this systematic review was improvement in symptom severity, which was measured using the International Restless Legs Syndrome (IRLS) rating scale. This scale comprises 10 questions that evaluate the frequency and intensity of symptoms, as well as their impact on daily life. Each question is scored from 0 to 4 based on perceived severity. The total score ranges from 0 to 40 points, where higher values indicate more severe symptoms. This scale is employed to monitor symptom progression and treatment response in RLS patients.

Secondary outcomes included variations in sleep quality measured by the Pittsburgh Sleep Quality Index (PSQI) and daily sleepiness assessed through the Epworth Sleepiness Scale (ESS). The first consists of 19 questions grouped into 7 components regarding sleep quality. Each component is scored from 0 to 3, with a total score ranging from 0 to 21. Higher scores indicate poorer sleep quality, with a threshold of 5 or more indicating poor sleep. The second one consists of eight questions that evaluate the likelihood of falling asleep in various everyday situations. Each question is scored from 0 to 3 based on the chance of dozing off, where 0 means “never” and 3 means “highly likely”. The total score ranges from 0 to 24, with higher scores indicating greater daytime sleepiness. 

### 3.4. Vitamin B6 and Magnesium Oxide

Jadidi et al. [37] developed a trial consisting of a combined intervention involving supplements and pharmacological therapy, comparing three groups. One control group would solely receive pramipexole, while the other two groups would additionally receive either vitamin B6 or magnesium oxide. The mean and standard deviation of sleep quality and disease severity at the beginning of the trial and throughout the first month following the intervention did not differ statistically between the three groups. However, after two months, even if all three groups attained relative progress, the mean score of sleep quality and the IRLS questionnaire differed significantly between the three assessments in all three groups (*p* < 0.05), with both intervention groups outperforming the control group. Mean PSQI and IRLS questionnaire scores among intervention groups were considerably lower in the magnesium oxide group compared to the vitamin B6 group, and this difference was statistically significant (*p* = 0.05).

### 3.5. Vitamin D

According to Wali et al. [38] findings, after 12 weeks of assessing the efficacy of vitamin D replacement, they were not able to observe a significant change in the RLS severity score (mean difference = 4.2; 95% CI −12.8 to +4.4, *p* = 0.32) compared to those in the placebo group. Even when the data analysis was restricted to only participants with vitamin D deficiency, this fact remained consistent. Even if they were not able to find any significant improvement with vitamin D supplementation, they noticed that the RLS severity score was significantly different between baseline and week 12 in the placebo group (*p* < 0.05), which persisted when analyzing just vitamin D deficient patients. This may have occurred due to a placebo effect, nonetheless, this effect was not observed in the vitamin D group, which raised concern about the possibility of causing harm to patients.

### 3.6. Oral Iron (Ferrous Sulfate)

Among the four articles included in this systematic review examining the use of oral iron as a therapeutic alternative for RLS treatment, two of them compared their efficacy with other pharmacological options, while the other two compared them with a placebo group. On one hand, when contrasting oral iron with pharmacological medications, Vishwakarma et al. [39] compared three different intervention groups that took either ropinirole, bupropion, or elemental iron along with folic acid, whereas Lee et al. [41] compared two groups receiving either pramipexole or ferrous sulfate as treatment for RLS. In the first case, IRLS scores differed significantly from the baseline visit to the last (*p* = 0.01) and the interaction between the time x treatment group was again significant (*p* < 0.001) showing an improvement with the therapy in all the groups. All three groups differed significantly from each other (*p* = 0.001), with the ropinirole group being the one that presented the lowest IRLS scores. When assessing RLS QoL, each group’s scores differed among all four visits (*p* = 0.002), but they were not able to find a significant difference among the RLS QoL scores between groups (*p* = 0.28). In the second case, IRLS scores were significantly lower than those at baseline in both groups (iron −9 ± 7, *p* < 0.001; pramipexole −9 ± 8, *p* = 0.001); however, it was not significantly different between the two groups. The Pramipexole group found a significant reduction in the mean PSQI score, meanwhile, no reduction was clear in the iron group. Nonetheless, group PSQI score changes were not significantly different, and ESS and BDI showed no significant change compared to baseline in either group.

On the other hand, when the comparison was made with a placebo group, Wang et al. [42] found mean decreases in IRLS scores after 12 weeks for iron and placebo groups were 10 ± 7 and 1 ± 6, respectively (*p* = 0.01). Although these changes were significant, when they compared dichotomized overall improvement in quality-of-life variables, they were not able to find significant differences between groups (*p* = 0.07). It is worth noting that both groups took vitamin C supplementation daily throughout the study. Conversely, in the study by Davis et al. [43], they did not find significant differences among iron and placebo groups in either primary or secondary outcomes.

### 3.7. Vitamin C and Vitamin E

Regarding the utilization of this supplement, two studies were encompassed in this systematic review: Rafie and Jafari’s [40], which compared a cohort of patients who were administered vitamin C with two other groups who received either pramipexole or a placebo, and Sagheb et al.’s [29], which divided patients into four groups based on whether they underwent intervention with vitamin C, vitamin E, a combination of both, or a placebo. Both studies assessed just RSL severity of symptoms and used IRLS scores for outcome analysis. In the first study, both vitamin C and Pramipexole groups had significantly improved IRLS scores before the intervention and were both significantly different from scores obtained in the placebo group. Despite that, IRLS scores were not significantly different between the two intervention groups (*p* = 0.77). The second one showed decrements in IRLS sum scores among all four groups; however, the reduction in IRLS sum scores was significantly higher in intervention groups rather than the placebo group (*p* < 0.001). Additionally, there were no variations in the decreases in IRLS sum scores among the treatment groups.

### 3.8. Valerian

Two articles that used valerian as an RLS intervention were included in this systematic review. Hajizadeh et al. [36] showed that both valerian and gabapentin groups showed significant decreases in RLS mean scores (*p* < 0.05), which was lower in the gabapentin group, especially in the first month. Moreover, even if both groups showed significant improvements in quality of sleep (*p* < 0.05), they were equally effective when comparing PSQI mean scores before and after the first and second interventions. In Cuellar and Ratcliffe’s [32] trial, they compared the efficacy of valerian with a placebo group, and even if both groups reported improvement in RLS symptom severity, sleep, and quality of life (IRLS, RLS QoL and PSQI scores), they could not find statistically significant differences. However, in a nested analysis comparing sleepy vs. nonsleepy participants, significant differences before and after treatment were found in sleepiness (*p* = 0.01) and RLS symptoms (*p* = 0.02). Moreover, a strong positive association between changes in sleepiness and RLS symptom severity was found (*p* = 0.006).

### 3.9. Quality Assessment

**Figure 2:**
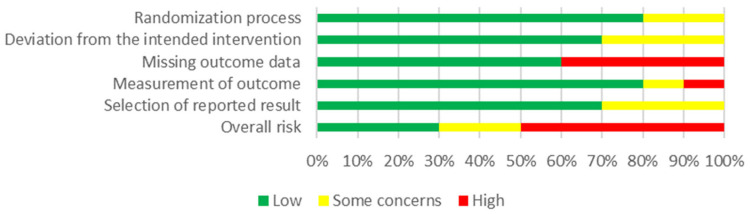
Risk of bias graph about each risk of bias domain presented as percentages across all included studies.

Among the ten included articles, five of them exhibited a “High” risk of bias, due to a high risk of missing the outcome data domain [32,36,39,41] or measurement of the outcome [43]. Two studies showed “Some concerns” of risk of bias, presenting some concerns in either deviation from the intended intervention or selection of reported results [37,42]. The remaining three articles showed a low risk of bias [29,38,40]. Figure 2 summarizes the risk of bias in the included studies following an assessment conducted by the authors through the algorithm of the RoB2 tool.

## 4. Discussion

This systematic review aimed to assess the impact of dietary supplementation on the clinical severity of patients with RLS. Supplements such as magnesium oxide and vitamin B6 showed significant improvements in sleep quality and RLS symptom severity compared to the control group, with magnesium demonstrating greater effectiveness. However, a moderate overall risk of bias was present, and specifically in the deviation from the intended interventions items. The vitamin D did not yield significant improvements, even in participants with vitamin D deficiency. Oral iron results were mixed, showing some symptom improvements but no significant differences compared to other treatments. Vitamins C and E also demonstrated positive effects, although differences between intervention and control groups were not always significant. Valerian showed benefits in sleep quality and symptom severity in some studies, although statistical significance was not consistently achieved across included studies.

Magnesium may play a role in the therapy of RLS, but its mechanism of action can currently only be speculated upon until reports about a consensus theory emerge [24,44]. One possible explanation could involve magnesium’s direct impact on the nervous system, as its inhibitory effect on neuronal excitability has long been recognized and is attributed to its physiological antagonism with calcium [45]. In the central nervous system, magnesium appears to play a regulatory role in the function of various neurotransmitters, including acetylcholine and gamma-aminobutyric acid [46]. Furthermore, there is evidence of its direct influence on the function of N-methyl-D-aspartate receptors [47].

The theory that magnesium may play a role in RLS is supported by findings from several studies, which have revealed lower levels of magnesium in patients with RLS compared to healthy control groups [13,48,49]. Furthermore, reports are linking the severity of RLS symptoms to lower magnesium levels [26,50]. However, blood magnesium levels may not be indicative of magnesium levels in muscle or the central nervous system (CNS) [24], as reflected in a study that reported no significant differences in blood or cerebrospinal fluid magnesium levels between RLS patients and the control group [51].

Only one randomized clinical trial examining magnesium supplementation was included, and according to their findings [37], scores on the IRLS and PSQI questionnaires were significantly better in the group receiving magnesium compared to those receiving vitamin B6 or placebo. It is worth noting that although this study yielded significantly positive results, available systematic reviews present conflicting outcomes. While one review positions magnesium as a potentially cost-effective and relatively safe option [24], another asserts that no solid conclusions can be drawn regarding its effectiveness [52], and yet another does not consider it a worthwhile option for treating RLS symptoms [53]. A recent open-label pilot study explored the use of magnesium citrate instead of magnesium oxide administered daily for eight weeks. The results showed a significant reduction in IRLS scores in the participants. In this sense, there is a need for placebo-controlled randomized clinical trials to explore the effects of this form of magnesium supplements [54].

Jadidi et al. [37] also addressed the use of vitamin B6 as a supplement to treat RLS. Although its efficacy was lower than that of the magnesium group, it significantly improved scores on the IRLS and PSQI questionnaires compared to the placebo group. The rationale for its use in these patients could be its influence on sleep, as studies have linked vitamin B6 intake with sleep quality [55], showing a reduction in nocturnal awakenings [56]. A lack of pyridoxine (vitamin B6) can lead to disrupted signaling between neurons, typically manifesting as muscle spasms and peripheral neuropathy; moreover, pyridoxal phosphate molecules are crucial for preserving enzymatic activity and muscle function [57].

Another supplement that could play an important role is vitamin D. A relationship between vitamin D and RLS has been hypothesized, as several studies have found significantly lower levels of 25-hydroxyvitamin D in the blood of RLS patients compared to the control group [58,59,60]. Some studies have also reported an inverse correlation between serum levels of 25-hydroxyvitamin D and the severity of RLS [59], and even greater severity of RLS in patients with 25-hydroxyvitamin D deficiency [60]. In this line, RLS was more frequent in patients with serum 25OHD levels < 20 ng/mL compared to those with serum 25OHD > 20 ng/mL. A decrease in RLS severity was reported in patients with RLS after vitamin D supplementation (28,000 IU/week) [61]. The reason why a deficiency in vitamin D could be associated with the development and clinical severity of RLS lies in the possibility that it may lead to dopaminergic dysfunction via the nigrostriatal pathway [58,62,63].

Only one trial addressing the use of vitamin D as a supplement was included. According to the findings of Wali et al. [38], patients who received vitamin D supplementation did not show significant changes in their average scores on the IRLS scale, a trend that persisted when analyzing only patients with vitamin D deficiency. Furthermore, significant differences were observed when comparing these scores with those of the placebo group, raising concerns about whether vitamin D could have a detrimental effect on patients.

Among the studies addressing oral iron supplementation, three reported significant improvements in IRLS scores [39,41,42], with progress significantly lower compared to ropinirole [39], similar to bupropion [39] or pramipexole [41], and significantly higher compared to a placebo group [42]. However, Davis et al. [43] did not show significant differences in any of the variables studied when compared to the placebo group. Among the potential causes of RLS, iron deficiency stands out as one of the most significant from a pathophysiological perspective [64]. This is based on clinical findings indicating that iron deficiency increases the frequency of RLS [65] and correlates with symptomatic severity [66,67,68]. Regional iron deficits in the brain appear to play a central role in the pathophysiology of RLS, which could instigate alterations in various neurotransmitter systems such as dopaminergic, glutamatergic, and adenosinergic pathways [62]. In contrast to magnesium, iron has shown reduced levels in cerebrospinal fluid in individuals with RLS [69], decreased concentrations in the substantia nigra through magnetic resonance imaging [70], and depleted iron stores in the substantia nigra observed in autopsy samples [71]. Therefore, even patients with normal blood ferritin levels may benefit from oral iron therapy [42], and unlike other medications aimed at treating symptoms of the disease, it could address a potential cause related to iron levels in the brain and normalize dopaminergic abnormalities [72]. However, according to the recommendation of the International Restless Legs Syndrome Study Group in their evidence-based clinical guidelines and consensus, oral iron therapy should only be considered in adults with serum ferritin levels below 75 µg/L [73].

The advantages of taking oral iron are limited by challenges in therapeutic compliance, often due to gastrointestinal discomfort [74], as well as absorption limitations [75]. Because of this, it is common to co-administer iron with vitamin C, as it enhances iron absorption by reducing ferric iron to ferrous iron through its antioxidant property, preventing re-conversion to ferric iron, and its potential to chelate iron thereby improving absorption [76]. Given that vitamin C enhances iron absorption, it was administered alongside iron in one of the studies included in this review [42]. Additionally, it has been used independently [29,40] or in combination with other supplements such as vitamin E [40] to treat RLS patients. Supplementation with vitamin C may also be effective as it enhances the activity of tyrosine hydroxylase in the brain [77], thereby promoting dopamine synthesis [78]. It is noteworthy that vitamins C and E act as potent antioxidants and could be considered options, as there is evidence indicating significantly elevated total oxidative status in RLS patients [79]. In this review, three studies used vitamin C, and one used vitamin E. In the study by Wang et al. [42] vitamin C was not used to treat symptoms but to enhance oral iron absorption. Rafie and Jafari [40] reported a significant improvement in IRLS scores in the vitamin C group compared to the placebo group, but not compared to the pramipexole group. Finally, Sagheb et al. [29] demonstrated a significant decrease in IRLS scores in the vitamin C + vitamin E, vitamin C, and vitamin E groups compared to the placebo group, but no significant differences were observed between the intervention groups. According to previous evidence, the total body content of vitamin C ranges from 300 to 2000 mg. The adequate dosage is relevant, given that high levels of vitamin C are shown to be maintained in cells and tissues, while low levels of vitamin C are in extracellular fluids and saliva [80]. Regarding vitamin E supplementation, the studies include α-tocopherol although mixed products containing other tocopherols and even tocotrienols are available [81].

The last dietary supplement studied in this systematic review was valerian, for which contradictory results were obtained in the two randomized clinical trials included. While Hajizadeh et al. [36] found significant improvements in mean scores on the IRLS and PSQI questionnaires compared to baseline values, Cuellar and Ratcliffe [32] did not find such differences, not even compared to a placebo group. These are the only studies we have been able to find addressing the efficacy of valerian in reducing the symptomatic severity of RLS, with several reviews stating that there is insufficient evidence to determine whether valerian is effective in reducing the symptomatic severity of RLS [23,82]. Regarding sleep quality, there are other studies demonstrating the efficacy of valerian compared to placebo [83]; furthermore, when compared to pharmacological therapy, its similar efficacy could suggest that it should be preferred as a first-line option given its herbal nature [36].

### Limitations

Despite the comprehensive nature of this review, several limitations must be acknowledged. The review included only ten randomized clinical trials, with a relatively small sample size that constrains the robustness of the conclusions drawn. Of these, five were evaluated as having a “high” risk of bias, indicating significant methodological flaws that could skew results and limit the reliability of the findings, while two were assessed as having “some concerns” regarding bias. Additionally, the studies demonstrated considerable heterogeneity in terms of inclusion and exclusion criteria and the specific supplements studied, which complicates the synthesis of results and the formulation of definitive conclusions. Most studies focused on specific supplements without providing comprehensive data across the entire spectrum of dietary supplements evaluated, thereby reducing the generalizability of the findings. Furthermore, many trials had a short follow-up period, which may not capture the long-term effects of supplement use on RLS symptoms, quality of sleep, or daytime sleepiness. 

While this systematic review provides an overview of the current research on dietary supplements for RLS, the findings should be interpreted with caution due to the limited number of high-quality randomized clinical trials, significant risk of bias in many studies, and variability in study designs and outcomes. Further high-quality, large-scale randomized clinical trials with rigorous methodologies are needed to establish the efficacy and safety of these supplements in the treatment of RLS.

## 5. Conclusions

Among the various supplements studied in this systematic review, conclusions vary depending on the supplement used. One study shows that magnesium and vitamin B6 supplementations alleviate RLS symptoms, with magnesium being superior in terms of efficacy, albeit with limited evidence. In contrast, vitamin D supplementation did not yield significant benefits, raising doubts about its efficacy and security. Oral iron supplementation seems to offer a promising avenue, especially for individuals with iron deficiency, although challenges remain in therapeutic compliance and absorption. Supplementation with vitamins C and E improved symptoms, likely due to their antioxidant properties. The effectiveness of valerian remains uncertain due to conflicting results and lack of evidence.

However, the interpretation of these findings is limited by several factors, including small sample sizes, short follow-up periods, methodological deficiencies, and heterogeneity among studies. Despite these limitations, this review underscores the importance of exploring alternative therapeutic options for RLS beyond traditional pharmacological interventions. There is a need for more high-quality randomized controlled trials with larger sample sizes and long-term studies to establish the true efficacy and safety of dietary supplements in managing RLS.
